# Estratégias de controle e atenção à tuberculose multirresistente: uma revisão da literatura

**DOI:** 10.26633/RPSP.2019.20

**Published:** 2019-02-06

**Authors:** Jaqueline Garcia de Almeida Ballestero, Mônica Cristina Ribeiro Alexandre d´Auria de Lima, Juliana Masini Garcia, Roxana Isabel Cardozo Gonzales, Amélia Nunes Sicsú, Fernando Mitano, Pedro Fredemir Palha

**Affiliations:** 1 Escola de Enfermagem de Ribeirão Preto Escola de Enfermagem de Ribeirão Preto Universidade de São Paulo (USP) Ribeirão PretoSP Brasil Universidade de São Paulo (USP), Escola de Enfermagem de Ribeirão Preto, Ribeirão Preto (SP), Brasil.; 2 Faculdade de Enfermagem Faculdade de Enfermagem Universidade Federal de Pelotas (UFPel) PelotasRS Brasil Universidade Federal de Pelotas (UFPel), Faculdade de Enfermagem, Pelotas (RS), Brasil.; 3 Escola Superior de Ciências da Saúde Escola Superior de Ciências da Saúde Universidade do Estado do Amazonas ManausAM Brasil Universidade do Estado do Amazonas, Escola Superior de Ciências da Saúde, Manaus (AM), Brasil.; 4 Faculdade de Ciências de Saúde Faculdade de Ciências de Saúde Universidade Lúrio Nampula Moçambique Universidade Lúrio, Faculdade de Ciências de Saúde, Nampula, Moçambique.

**Keywords:** Tuberculose resistente a múltiplos medicamentos, políticas públicas de saúde, administração em saúde pública, colaboração intersetorial, serviços de saúde, Tuberculosis, multidrug resistant, public health policy, public health administration, intersectoral collaboration, health services, Tuberculosis resistente a múltiples medicamentos, políticas públicas de salud, administración en salud pública, colaboración intersectorial, servicios de salud

## Abstract

**Objetivo.:**

Identificar estratégias de controle da tuberculose multidrogarresistente (TBMDR) e de atenção aos pacientes acometidos.

**Métodos.:**

Foi realizada uma revisão integrativa da literatura mediante pesquisa em três bases de dados da área da saúde (LILACS PubMed e CINAHL) e uma multidisciplinar (Scopus). Foram selecionados artigos originais que apresentassem estratégias utilizadas para operacionalização da atenção ao doente de TBMDR, publicados em inglês, espanhol ou português de 2006 a 2016. As informações coletadas foram organizadas segundo as estratégias para operacionalização da TBDMR identificadas pelos pesquisadores, que foram agrupadas em categorias temáticas.

**Resultados.:**

Com base em uma amostra de 13 artigos, foram identificadas quatro categorias: a) DOTS-plus: reorganização dos serviços de saúde, aprimoramento das estruturas locais, padronização de fluxos e condutas profissionais, oferta de tratamento diretamente observado; b) descentralização do serviço: aproximação entre profissionais de saúde e doentes em tratamento, prioritariamente em locais com alta carga da doença; c) uso de ferramentas de comunicação: software *e ligações telefônicas que permitiram a* realização de consultoria com especialistas e/ou otimização da assistência dentro das equipes multiprofissionais; d) proteção social dos doentes: criação de mecanismos que forneceram apoio emocional, social e/ou econômico aos doentes em tratamento, fortalecendo a adesão à terapia medicamentosa.

**Conclusões.:**

Foram identificadas diversas estratégias para além dos aspectos farmacológicos, corroborando a ideia de que o controle da TBMDR exige mecanismos que possibilitem uma atenção integral e condizente com as peculiaridades e potencialidades dos diferentes cenários onde a doença ocorre.

## Abstract (en)

**Objective.:**

To identify control and patient management strategies for multidrug-resistant tuberculosis (MDR-TB).

**Methods.:**

An integrative review of the literature was performed through research in three health databases (LILACS, PubMed and CINAHL) and one multidisciplinary database (Scopus). Original articles published in English, Spanish or Portuguese, from 2006 to 2016, describing strategies to implement MDR-TB patient care, were included. The information collected was organized according to the strategies identified by the investigators, which were grouped into theme categories.

**Results.:**

Based on a sample of 13 articles, four categories were identified: a) DOTS-plus: reorganization of health services, improvement of local structures, standardization of professional protocols and behaviors, provision of directly observed treatment; b) service decentralization: bringing health professionals closer to patients, especially in areas with high disease burden; c) use of communication tools: software and telephone calls that allowed consultations with specialists and/or optimization of care within multiprofessional teams; d) social protection of patients: establishment of mechanisms to provide emotional, social and/or economic support to patients under treatment, strengthening adherence to drug therapy.

**Conclusions.:**

Several strategies were identified beyond pharmacological measures, supporting the idea that the control of MDR-TB requires mechanisms that allow comprehensive care, consistent with the peculiarities and potentialities of the different scenarios where the disease occurs.

## RESUMEN

**Objetivo.:**

Identificar estrategias de control de la tuberculosis multirresistente (TBMR) y de atención a los pacientes afectados.

**Métodos.:**

Se realizó una revisión integrativa de la literatura en tres bases de datos del área de la salud (LILACS, PubMed y CINAHL) y en uma base de datos multidisciplinaria (Scopus). Se seleccionaron artículos originales que registraran las estrategias utilizadas para la atención al paciente con TBMR, publicados en inglés, español o portugués entre 2006 y 2016. Se organizaron los datos de acuerdo a las estrategias de implementación de la TBMR identificadas por los investigadores, que fueron agrupadas en categorías temáticas.

**Resultados.:**

Con base en una muestra de 13 artículos, se identificaron cuatro categorías: a) DOTS-plus: reorganización de los servicios de salud, mejoramiento de la estructura local, estandarización de protocolos y comportamientos profesionales, administración de tratamiento supervisado; b) descentralización del servicio: aproximación entre profesionales de la salud y pacientes en tratamiento, principalmente en los lugares con alta carga de la enfermedad; c) uso de herramientas de comunicación: programas informáticos y llamadas telefónicas que permitieran la realización de consultas con especialistas o la optimización de la asistencia dentro de los equipos multiprofesionales; d) protección social de los pacientes: creación de mecanismos que proporcionaron apoyo emocional, social o económico a los pacientes en tratamiento, fortaleciendo la adhesión al tratamiento farmacológico.

**Conclusiones.:**

Se identificaron diversas estrategias que van más allá de los aspectos farmacológicos, lo que corroboró la idea de que el control de la TBMR exige mecanismos que posibiliten una atención integral de acuerdo a las particularidades y potencialidades de los diferentes escenarios donde ocurre la enfermedad.

Apesar de o esquema medicamentoso preconizado e padronizado para tratamento da tuberculose em grande parte dos países estar relacionado à cura da maioria dos casos novos, a ocorrência da resistência às drogas antituberculosas agrava a problemática da doença (1). Especialmente grave é a resistência conjunta aos dois principais fármacos do tratamento (rifampicina e isoniazida) – caracterizando a tuberculose multidrogarresistente (TBMDR) (2).

O desenvolvimento da TBMDR é um fenômeno complexo que envolve diversos fatores. Além do uso inadequado dos esquemas prescritos por parte dos doentes, as dificuldades na organização da assistência à tuberculose, com falta de normatização e padronização dos tratamentos ou ausência de treinamento das equipes e de acompanhamento da evolução da terapia, favorecem o desenvolvimento de formas resistentes (3).

Atualmente, 30 países concentram 95% dos casos mundiais de TBMDR, entre os quais encontram-se Brasil e Peru, como representantes das Américas (4). Porém, acredita-se que os números oficiais reportados à Organização Mundial da Saúde (OMS) estejam subestimados, já que 3,9% dos casos novos de tuberculose e 21% dos doentes previamente tratados desenvolvem TBMDR. Dessa forma, de 250 000 possíveis casos de TBMDR, somente 150 000 foram notificados em 2016 (5). Sendo assim, as taxas totais de cura dos casos multirresistentes são irrisórias e insuficientes para conter a epidemia (5).

As consequências da TBMDR são desastrosas, ocasionando uso de medicamentos menos eficazes e mais tóxicos e maior ocorrência de efeitos adversos graves e potencialmente fatais (5-7). Essa forma de tuberculose está associada a uma elevada taxa de mortalidade; também torna o tratamento limitado, mais longo e com custo orçamentário mais elevado, tanto para as pessoas acometidas quanto para os sistemas de saúde (8-10).

O manejo da TBMDR é muito dependente das estruturas locais, que, muitas vezes, não seguem as recomendações da OMS (11). Além dos esquemas terapêuticos, é preciso garantir o acesso dos pacientes à atenção especializada, oferecer alternativas às dificuldades socioeconômicas dos doentes e fortalecer a comunicação entre os diversos serviços de saúde envolvidos na assistência, entre outras medidas (12). Dessa forma, o objetivo do presente estudo foi identificar na literatura as estratégias de controle da TBMDR e atenção às pessoas acometidas por essa condição.

## MATERIAIS E MÉTODOS

Foi realizada uma revisão integrativa da literatura. A questão norteadora foi estruturada por meio da estratégia PICO (13, 14): a população (P) foi definida como pacientes com TBMDR; a intervenção (I) foi definida como as estratégias para tratamento da TBMDR; o item controle/comparador (C) não se aplicou no presente caso; e o desfecho (O) foi definido como os resultados da operacionalização das estratégias. Assim, foram buscados estudos que respondessem à questão norteadora “quais são as estratégias utilizadas para operacionalização da atenção ao doente de tuberculose multidrogarresistente?”.

A revisão integrativa foi desenvolvida em seis etapas: estabelecimento da questão da revisão, busca na literatura, extração dos dados dos estudos primários, avaliação dos estudos primários incluídos na revisão, análise e síntese dos resultados e apresentação da revisão (15). Foram pesquisadas três bases de dados da área da saúde – LILACS (http://lilacs.bvsalud.org/), PubMed (https://www.ncbi.nlm.nih.gov/pubmed/) e CINAHL (https://www.ebsco.com/products/research-databases/cinahl-complete) – e uma multidisciplinar – Scopus (https://www.scopus.com/search/form.uri?display=basic). A estratégia de busca utilizou uma combinação de descritores em ciências da saúde (DeCS), *Medical Subject Headings* (MeSH) e títulos CINAHL. Os MeSH foram usados como termos de busca na base multidisciplinar Scopus. Os descritores foram combinados da seguinte maneira: (Tuberculosis, Multidrug-Resistant OR Tuberculosis, Multidrug Resistant OR Tuberculosis, MDR OR Multi-Drug Resistant Tuberculosis OR Tuberculosis, Drug-Resistant) AND (Health services research OR systems integration OR community health services OR administration, public health OR health policy OR accountable care organizations OR disease management OR regional medical programs OR centralized hospital service) AND (Treatment Outcome OR Clinical Effectiveness OR Patient-Relevant Outcome OR Clinical Efficacy OR Treatment Effectiveness OR Rehabilitation Outcome).

Para a seleção dos artigos, os critérios de inclusão foram: ser artigo original que apresentasse as estratégias utilizadas para operacionalização da atenção ao doente de TBMDR e ter sido publicado em inglês, espanhol ou português de 1º de janeiro de 2006 a 31 de dezembro de 2016, período considerado adequado para levantamento de estratégias atualizadas. Foram excluídas publicações secundárias, estudos de caso, estudos de revisão, editoriais e artigos de opinião.

As buscas e a seleção dos estudos elegíveis foram realizadas por dois revisores (JAGB, MCRAAL) de forma independente. As buscas dos estudos nas bases de dados foram realizadas em um único dia do mês de agosto de 2017. O gerenciador de referências EndNote X7 foi utilizado para organizar os estudos a partir de cada base de dados. Sendo assim, os dois autores revisaram todos os 1 247 artigos inicialmente identificados em 1 dia. As listas de referências dos artigos lidos na íntegra também foram examinadas. Em caso de discordância entre os revisores, os mesmos discutiram conjuntamente e chegaram a um consenso. Na impossibilidade de consenso, um terceiro revisor (NAS) foi consultado.

A utilização do EndNote X7, posteriormente, auxiliou no agrupamento dos artigos em categorias. Os estudos selecionados para inclusão foram lidos na íntegra, sendo extraídas as seguintes informações, a partir de um roteiro previamente estruturado pelos autores: título, ano de publicação, local de estudo, objetivo, método, principais resultados e contribuições para a presente investigação. Essas informações foram organizadas segundo as estratégias para operacionalização da TBDMR identificadas pelos pesquisadores. Essas estratégias foram agrupadas em categorias temáticas criadas e nomeadas pelos pesquisadores.

## RESULTADOS

**FIGURA 1:**
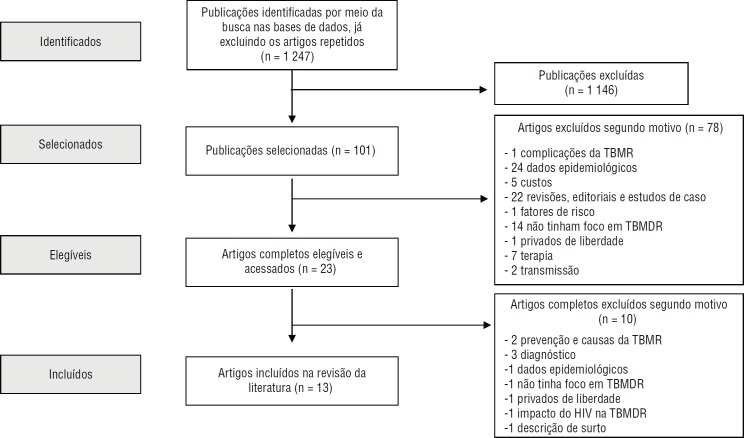
Fluxograma de seleção de estudos sobre estratégias para controle da tuberculose multirresistente, 2006 a 2016

Foram identificados 1 247 artigos, já excluídas desse total as publicações repetidas. Depois da aplicação dos critérios de inclusão e exclusão, 101 títulos foram selecionados para leitura de resumos e 23 artigos foram lidos na íntegra. Nenhum artigo foi incluído a partir da revisão manual das referências contidas nos artigos. Finalmente, 13 artigos compuseram a amostra analisada (figura 1).

A amostra foi constituída por estudos publicados de 2007 a 2015, todos em inglês. Três estudos foram desenvolvidos na África do Sul (16-18), dois na Índia (19, 20), um no Peru (21) e um no Peru e outros países (Haiti, Paquistão, Nepal, Tajiquistão, Indonésia, Quênia e Botsuana) (22). Um estudo foi identificado também em cada uma das seguintes regiões: Filipinas (23), Rússia (24), República Democrática do Congo (25), Reino Unido (26), Equador (27) e Leste Europeu (28).

A maioria das investigações foi descritiva, tipo relato de experiência, de abordagem quantitativa. O tamanho da amostra variou de 3 a 1 294 doentes de TBMDR, sendo que dois estudos analisaram as mensagens inseridas em *software –* que consistiam em resultados de exames (baciloscopia, cultura de escarro, teste de sensibilidade às drogas), terapia medicamentosa prescrita, medicamentos dispensados, eventos adversos à terapêutica e outras informações relevantes para manejo dos casos. A tabela 1 mostra os tipos de estudos, tamanho das amostras, período de realização e as estratégias analisadas.

Para apresentação dos resultados e discussão dos achados, os estudos foram agrupados em quatro categorias conforme as estratégias identificadas: DOTS-plus; descentralização do serviço; uso de ferramentas de comunicação; e proteção social dos doentes.

### Estratégia DOTS-plus

Esta categoria corresponde a artigos que estudaram a implantação da estratégia de tratamento diretamente observado *(Directly Observed Therapy, Short-Course*-*plus,* DOTS-plus) como forma de reorganização dos serviços de saúde. Integram este grupo quatro estudos.

O primeiro estudo descreveu a implementação de DOTS-plus no cenário filipino e mostrou aumento da adesão ao tratamento. Os autores apontam como fatores principais o treinamento da equipe, a construção e supervisão de laboratórios, a padronização de condutas e o desenvolvimento do cuidado centrado no doente, sendo pontos fundamentais: tratamento diretamente observado no domicílio, atenção psicossocial e envolvimento de parceiros comunitários que tiveram TBMDR e se curaram (23).

A segunda publicação descreveu a implantação de ­DOTS-plus em Déli, na Índia. O tratamento diretamente observado dos doentes se deu em unidades de saúde próximas dos domicílios, com apoio de um familiar para supervisão da dose noturna. No entanto, o estudo evidenciou uma elevada ocorrência de óbito (29%) no primeiro mês de tratamento devido ao atraso no diagnóstico de resistência e início de terapia oportuna (19).

Já o estudo desenvolvido na Rússia apontou as ações empreendidas e sua correspondência aos componentes do DOTS-plus. Destacam-se a realização do tratamento diretamente observado no serviço de saúde ou no domicílio do doente e a disponibilidade de auxílio transporte e/ou alimentação. Com isso, os resultados apontam adequadas taxas de desfecho favorável do tratamento (24).

Outra pesquisa realizada na Índia descreveu os resulta­dos do tratamento após implantação da estratégia DOTS-plus em uma área predominantemente rural. No primeiro mês, o acompanhamento dos doentes aconteceu em uma instituição hospitalar. Depois da internação, passaram a ser tratados em um único serviço de referência, com o tratamento diretamente observado sendo realizado pela atenção primária à saúde. Mostrou-se a importância da proximidade a um profissional com perfil adequado para fortalecer a adesão e melhorar a capacidade de gerenciamento das reações adversas (20).

Todos os estudos desta categoria mostraram uma adequada implementação da estratégia em questão. Além de ressaltarem o tratamento diretamente observado como eixo central da estratégia, ressaltaram a importância da criação de medidas para acompanhar o doente de forma mais próxima aos serviços de saúde e considerando outros personagens para a efetivação do tratamento diretamente observado, como agentes comunitários, familiares e pessoas que já haviam passado por processo de adoecimento por TBMDR.

**TABELA 1:** Descrição dos estudos identificados na revisão integrativa sobre estratégias para controle da tuberculose multirresistente, 2006 a 2016

Autor (referência)	Delineamento	Tamanho da amostra	Período de realização do estudo	Estratégia
Quelapio et al. (23)	Relato de experiência, abordagem quantitativa	1 294 doentes tratados e 449 profissionais treinados	Três períodos: – DOTS-plus piloto: 1999 a 2003 – Fase de expansão: 2003 a 2006; – Treinamento de recursos humanos: 2007 a 2008	Implantação do DOTS-plus em parceria público-privada
Singla et al. (19)	Relato de experiência, abordagem quantitativa	126 doentes	Janeiro de 2002 a dezembro de 2006	Implantação do DOTS-plus em área urbana
Keshavjee et al. (24)	Estudo descritivo, abordagem quantitativa	244 doentes	2007 a 2008	Implantação do DOTS-plus em local com grande população privada de liberdade
Thomas et al. (20)	Relato de experiência, abordagem quantitativa	66 doentes da zona rural	Maio de 1999 a dezembro de 2003	Implantação do DOTS-plus em local de grande população rural
Brust et al. (16)	Estudo descritivo, abordagem quantitativa	80 doentes de TBMDR; 66 coinfectados pelo HIV	Fevereiro de 2008 a abril de 2010	Descentralização da assistência em local de grande população rural e alta taxa de coinfecção pelo HIV
Loveday et al. (17)	Estudo descritivo-analítico, coorte, prospectivo, abordagem quantitativa	860 doentes de TBMDR; 419 seguidos em serviços descentralizados e 441 no Hospital Centralizado	Julho de 2008 a novembro de 2009	Descentralização da assistência para serviço de base comunitária
Heller et al. (18)	Estudo descritivo-analítico, experimental, tipo antes e depois, abordagem quantitativa	107 doentes	Comparação entre dois períodos: março a dezembro de 2008 vs. janeiro de 2001 e fevereiro de 2008	Descentralização da assistência para serviço de base comunitária
Shanks et al. (25)	Relato de experiência, abordagem quantitativa	3 doentes	2006	Descentralização da assistência em uma área de conflito; médicos clínicos recebiam apoio de especialistas em tuberculose
D’Ambrosio et al. (28)	Relato de experiência, abordagem quantitativa	Dados de 70 doentes inseridos no *software*	2014	Uso de *software* para comunicação entre o médico clínico e os especialistas em tuberculose (casos mais complexos)
Jordan et al. (26)	Estudo descritivo, abordagem quantitativa	71 doentes e 33 profissionais de saúde	Janeiro de 2008 a dezembro de 2009	Uso de *software* para comunicação entre o médico clínico e diversas especialidades envolvidas no controle da TBMDR
Fraser et al. (22)	Relato de experiência	Não se aplica – descreveu registros em *software*, não doentes	2001 a 2007	Uso de dois programas para comunicação entre os profissionais
Sripad et al. (27)	Estudo descritivo, abordagem qualitativa	97 doentes	2011	Transferência direta de valor em dinheiro para doentes que aderiram ao tratamento
Acha et al. (21)	Estudo analítico, abordagem qualitativa, com uso da etnografia	285 doentes	1999 a 2004	Grupo de apoio psicossocial para os doentes

### Descentralização do serviço

Como descrito anteriormente, uma importante ação do DOTS-plus é favorecer a proximidade entre profissionais e doentes. Nesse sentido, a segunda estratégia de controle de TBMDR compreende quatro estudos que abordaram a reorganização dos serviços e fluxos entre serviços e profissionais por meio da descentralização da assistência em países com alta carga da doença: África do Sul (16-18) e República Democrática do Congo (25). Além disso, foi estudado o impacto da transição do modelo de hospitalização para o de base comunitária, com atenção prestada ambulatorialmente.

O primeiro desses estudos descreve a reordenação da atenção voltada para TBMDR e HIV. Os doentes foram seguidos pelos mesmos profissionais de saúde: médicos, agentes comunitários de saúde (ACS) e enfermeiros (que realizavam a visita domiciliar e o tratamento diretamente observado). Um membro da família ou amigo realizavam o “suporte ao tratamento”. Todos os envolvidos recebiam treinamento sobre a doença a fim de melhorar a adesão e diminuir o estigma. Essa reordenação da atenção possibilitou uma taxa de sucesso no tratamento de 77% (considerando os casos curados e os que ainda estavam em tratamento) e, mesmo na presença de efeitos adversos, nenhum doente necessitou interromper a terapia (16).

No segundo artigo, compararam-se duas modalidades de tratamento existentes na África do Sul: o centralizado (âmbito urbano) e o descentralizado (perto das comunidades rurais e em diferentes áreas das províncias participantes). Os médicos foram treinados para iniciar e conduzir os tratamentos, sendo os casos mais complexos encaminhados para um hospital de referência. Foram criadas diretrizes para o tratamento considerando as realidades locais. Ao final, considerando as taxas de conversão da cultura e de cura, o tratamento descentralizado mostrou-se com melhor desempenho (17).

O terceiro artigo sul-africano verificou a alteração do *locus* de cuidado do hospital para o ambulatório (descentralizado). Houve melhorias nos indicadores de controle da doença: diminuição do tempo médio de início do tratamento específico para TBMDR, do tempo médio de negativação baciloscópica e do tempo médio de conversão da cultura. Porém, quanto ao desfecho do tratamento, não foi observada diferença estatisticamente significativa. Mesmo assim, os pesquisadores acreditam que o tratamento ambulatorial, descentralizado, pode ser uma alternativa viável naquele contexto (18).

Já o quarto estudo foi realizado em uma área de conflito na República Democrática do Congo, onde foi implantado um programa de tratamento para TBMDR. A parceria com a Bélgica possibilitou a realização de exames laboratoriais e de consultoria junto aos especialistas para a condução de casos, via telefone. O atendimento dos três doentes foi guiado por um protocolo simplificado: internação na fase intensiva; acompanhamento ambulatorial na fase de manutenção com tratamento diretamente observado diário, visitas mensais de médicos e enfermeiros e realização de cultura de escarro a cada 4 meses. A normatização de condutas e o contato direto com o laboratório melhorou a viabilidade do modelo, que até o momento de publicação do estudo era exitoso (25).

Os estudos mostraram que a descentralização dos serviços e as ações em saúde estiveram relacionadas à maior aproximação entre profissionais e doentes em tratamento, e também à implementação de protocolos/diretrizes. Há indícios de que a boa comunicação com a rede laboratorial está relacionada ao melhor estabelecimento da terapia, seguimento e definição de cura.

### Uso de ferramentas de comunicação

Assim como aparece no estudo abordado na categoria anterior (25), instrumentos que assegurem a comunicação entre especialistas e profissionais próximos aos doentes podem ser necessários para operacionalização da assistência. Frente a isso, esta categoria compreende três artigos cujo objetivo foi analisar o uso de ferramentas para viabilizar a comunicação como estratégia de melhoria da atenção.

Na primeira publicação é descrita a implantação do *Electronic TB Consilium* no Leste Europeu. Essa plataforma procurou garantir mecanismos de comunicação que permitissem a consultoria médica em casos complexos (coinfecção tuberculose-HIV, TBMDR e tuberculose em crianças). Como impactos do uso dessas ferramentas destacam-se: melhor gestão dos casos; impedimento ao desenvolvimento de resistência a outros medicamentos; monitorização e avaliação da condução das medidas de controle; identificação dos desafios enfrentados no tratamento; e apoio aos clínicos na tomada de decisões. As ferramentas foram incorporadas aos treinamentos da OMS e disponibilizada em vários idiomas, com planos de expansão e incorporação de um maior painel de especialistas (28).

Com intuito semelhante, foi desenvolvido no Reino Unido o *software Advisory Service MDR-TB*, com intuito de interligar equipes médicas centralizadas e especializadas em TBMDR a clínicos que prestavam assistência direta aos doentes. A maioria dos profissionais usuários do *software* sentiu-se confortável no envio de dados do doente, referiu satisfação com as opiniões obtidas junto aos especialistas e considerou o contato útil para escolha da terapia. Acredita-se que o uso dessa ferramenta repercutiu em melhoras quanto ao prognóstico dos casos, melhor disposição de dados e agilidade na comunicação entre os profissionais envolvidos (26).

No último artigo são descritas duas ferramentas peruanas de comunicação entre os diferentes profissionais pertencentes à mesma equipe para integrar os cuidados: o prontuário eletrônico e a plataforma OpenMRS-TB. O prontuário eletrônico possibilitou previsão e gestão do estoque de fármacos e redução do atraso nos resultados de exames laboratoriais. Já com a plataforma OpenMRS-TB, uma versão ampliada do prontuário eletrônico para uso móvel, observaram-se dificuldades para superação de fragmentação no fornecimento e uso das informações, principalmente no que diz respeito à interlocução com outras plataformas, mostrando necessidade de modificações (22).

A condução das ações para atenção à TBMDR é uma atividade complexa, que envolve profissionais de vários núcleos de competência. Logo, em diferentes momentos, é necessário garantir a comunicação efetiva entre os diversos atores e serviços de saúde envolvidos.

### Proteção social aos doentes

Integram esta categoria duas publicações que tiveram por objetivo investigar estratégias para melhorar a atenção pela criação de mecanismos que forneceram apoio emocional, social e/ou econômico aos doentes, contribuindo para a adesão à terapia medicamentosa. Essas estratégias incluem incentivos financeiros diretos, apoio alimentar, construção de moradias, apoio psicossocial e outras.

O primeiro estudo foi conduzido no Equador e investigou o impacto do fornecimento de benefício financeiro, ou seja, a transferência direta de um valor em dinheiro equivalente a US$ 240/mês aos doentes que aderiram ao tratamento. Essa iniciativa resultou na diminuição da não adesão (26,7% para 9,5%). As críticas mais frequentes foram de atrasos no recebimento dos valores. Os autores concluem que o programa de incentivo monetário aliviou o fardo econômico daqueles doentes, sendo o principal uso do dinheiro relatado para compra de alimentos (27).

O segundo estudo foi desenvolvido no Peru e versa a respeito do trabalho da organização não governamental *Socios en Salud* para fortalecimento da atenção à TBMDR na cidade de Lima. Acha et al. (21) descrevem e analisam o impacto de grupos de apoio psicossocial. Participaram dos grupos de apoio os doentes em tratamento e seus familiares, pessoas com história de TBMDR já atendidas pelo grupo, agentes comunitários de saúde, enfermeiros, assistentes sociais e psiquiatra. Doentes com sintomas psiquiátricos graves e/ou com cultura positiva recebiam terapia individual. A participação no grupo era voluntária e as atividades realizadas incluíram reuniões em grupo, excursões recreativas, celebrações simbólicas e oficinas periódicas para as famílias.

## DISCUSSÃO

Os resultados desta revisão evidenciaram que o controle da TBMDR não deve se esgotar na oferta de medicação e/ou uso de novas drogas, mas sim, deve incluir diferentes ações que propiciem a atenção ampliada ao doente em tratamento. A implantação da atenção ambulatorial para acompanhamento dos doentes, como ocorrido na África do Sul (16, 18), é fundamental para organizar a assistência, visto que o ambiente hospitalar favorece a disseminação de bacilos (29) e acarreta mais sofrimento psíquico às pessoas hospitalizadas (30).

A principal vantagem do acompanhamento ambulatorial apontada nos estudos analisados refere-se à possibilidade de proximidade entre equipes e doentes e, consequentemente, maior chance de êxito do tratamento (16, 24, 25). Alguns estudos apontaram para a melhoria da atenção após a descentralização dos serviços, especialmente em contextos com alta carga da doença (17, 20, 23). Além disso, descrevem a obtenção de bons resultados quando ocorreu a adequada distribuição dos pontos de assistenciais em locais estratégicos.

Outro aspecto de destaque refere-se à possibilidade de realização do tratamento diretamente observado na unidade de saúde e no domicílio, favorecendo a participação do familiar (19, 20, 23-25). Tais dados corroboram os achados de outra pesquisa que analisou os significados do processo de adoecimento por tuberculose e encontrou dificuldades de adesão ao tratamento nos doentes que não tiveram apoio familiar, ao passo que o suporte da equipe de saúde e o bom relacionamento com os profissionais ajudaram o doente a conviver com condição de saúde (31).

Os achados indicam a necessidade de atuação em várias frentes profissionais e comunitárias, envolvendo familiares, amigos e doentes já tratados para TBMDR. A atenção deve ser pautada pelo treinamento adequado e contínuo para acompanhamento dos casos pelas equipes e para o suporte social e emocional por parte da comunidade em que estão inseridos os doentes.

Ressalta-se, também, a importância de recursos financeiros ou benefícios para transporte e alimentação (21, 23, 24, 27). Além de acometer grande número de pessoas em situação de vulnerabilidade, a tuberculose acarreta elevados custos aos doentes, dificultando ou inviabilizando o trabalho e repercutindo em novos gastos para manutenção do tratamento. Tais custos são ainda mais pesados nos casos multirresistentes (32). Dessa forma, para o enfrentamento de uma condição de saúde tão complexa, é necessária uma atenção voltada para o bem-estar dos indivíduos, centrada nos doentes e no trabalho cooperado e complementar de profissionais e comunidade, ao encontro da estratégia atual da OMS (33).

Em um aspecto mais amplo, é vital a criação de mecanismos específicos que estabeleçam a discriminação positiva dessas pessoas (34) e que gerem mudanças na estrutura da dinâmica social e política, institucionalizando a proteção social de forma estável e irreversível (35). Logo, os benefícios sociais e econômicos concedidos aos doentes de TBMDR estariam ancorados em políticas concretas e permanentes nos diferentes cenários, direcionadas às especificidades desses pacientes, incluindo alimentação, transporte e demais aspectos.

Devido ao complexo manejo da TBMDR, é importante que a equipe esteja capacitada e que realize as ações de atenção com condutas padronizadas, lançando mão de diretrizes e protocolos para o tratamento, considerando a realidade de cada local (17, 23, 25). A adoção de condutas padronizadas e apoiadas em evidências científicas está relacionada a coordenação das ações profissionais, redução da ocorrência de falhas assistenciais, otimização dos gastos, resultando em maior eficiência econômica dos sistemas de saúde, e contribuição para a educação permanente das equipes (36).

Os trabalhos analisados mostraram a importância do uso de ferramentas eletrônicas, como *software*, ou mesmo de contato via telefone, que aproximem os especialistas da população atendida, visto que algumas realidades não contavam com médicos especializados na condução de casos de TBMDR. Ademais, os dispositivos tecnológicos auxiliam na gestão e previsão dos fármacos, sendo a oferta de medicamentos um importante pilar em todas as políticas de controle da tuberculose (12, 33, 37).

Tendo em vista a complexidade da TBMDR, é muito importante lançar mão de estratégias que auxiliem na atenção ao doente. Nesse sentido, esta revisão examinou diferentes estratégias fortalecedoras, inclusive medidas que enfatizam a organização adequada dos serviços e sistemas de saúde, gestão do cuidado, aparatos tecnológicos e envolvimento de vários atores sociais e profissionais, a fim de superar as limitações dos diferentes cenários.

As limitações do estudo estão relacionadas ao fato de a maioria dos artigos analisados terem utilizado o método descritivo, em sua maior parte relatos de experiências ocorridas em cenários específicos. A literatura carece de estudos randomizados ou mesmo melhor delineados do que os incluídos na presente revisão.

## Conclusões

Foi identificada uma grande gama de estratégias para além dos aspectos farmacológicos, corroborando a ideia de que o controle da TBMDR exige mecanismos que possibilitem uma atenção ampliada e condizente com as peculiaridades e potencialidades dos diferentes cenários. Assim, os aspectos destacados nesta revisão fornecem subsídios para a otimização dos programas de controle à doença nas Américas e no mundo.

Sobretudo, destacam-se questões de padronização de condutas, que permearam a estratégia de implantação do DOTS-plus nos diferentes contextos: normatização dos fluxos e manejo do tratamento; realização do tratamento diretamente por profissional de saúde treinado, sensibilizado e com perfil adequado; relevância de um apoiador para o tratamento; e treinamento adequado da equipe de saúde. Essas questões permearam também a estratégia de descentralização da assistência em contextos com alta carga da doença, somadas à preferência por seguimento ambulatorial, flexibilizado as peculiaridades dos contextos locais, com orientação de doente e familiares. Além disso, destacaram-se a integração dos profissionais e serviços de saúde por meio de estratégias de comunicação, assim como as estratégias que buscavam proteção social dos doentes, com grupos de apoio, combate ao estigma e estabelecimento de parcerias nacionais e internacionais, aliando os setores público e privado.

## Contribuição dos autores.

JGAB concebeu o estudo. JGAB e MCRAAL coletaram, analisaram e discutiram os dados. JMG, RICG, ANS, PFP e FM analisaram e auxiliaram na discussão dos dados. JGAB e MCRAAL redigiram o manuscrito. Todos os autores aprovaram a versão final do manuscrito.

## Conflitos de interesse.

Nada declarado pelos autores.

## Financiamento.

O presente trabalho foi realizado com apoio da Coordenação de Aperfeiçoamento de Pessoal de Nível Superior - Brasil (CAPES) - Código de Financiamento 001.
